# Augmentation of EMDR with multifocal transcranial current stimulation (MtCS) in the treatment of fibromyalgia: study protocol of a double-blind randomized controlled exploratory and pragmatic trial

**DOI:** 10.1186/s13063-021-05042-w

**Published:** 2021-01-29

**Authors:** I. Gardoki-Souto, O. Martín de la Torre, B. Hogg, D. Redolar-Ripoll, A. Valiente-Gómez, L. Martínez Sadurní, J. M. Blanch, W. Lupo, V. Pérez, J. Radua, B. L. Amann, A. Moreno-Alcázar

**Affiliations:** 1 Centre Forum Research Unit, Institut de Neuropsiquiatria i Addiccions, Parc de Salut Mar, C/ Llull 410, 08019 Barcelona, Spain; 2 Hospital del Mar Medical Research Institute (IMIM), Barcelona, Spain; 3 Department of Psychiatry and Forensic Medicine, Universitat Autònoma de Barcelona (UAB), Barcelona, Spain; 4 Cognitive NeuroLab, Universitat Oberta de Catalunya (UOC), Barcelona, Spain; 5 Centro de Investigación Biomédica en Red Salud Mental (CIBERSAM), Madrid, Spain; 6 Institut de Neuropsiquiatria i Addiccions (INAD), Parc de Salut Mar, Barcelona, Spain; 7 Service of Rheumatology, Parc de Salut Mar, Barcelona, Spain; 8 Institut d’Investigacions Biomèdiques August Pi i Sunyer (IDIBAPS), Barcelona, Spain; 9 Karolinska Institutet, Solna, Sweden; 10 King’s College, London, England

**Keywords:** Fibromyalgia, Eye movement desensitization and reprocessing, Multifocal transcranial current stimulation, Psychological trauma, Pain, Randomized controlled trial

## Abstract

**Background:**

Fibromyalgia (FM) is a generalized, widespread chronic pain disorder affecting 2.7% of the general population. In recent years, different studies have observed a strong association between FM and psychological trauma. Therefore, a trauma-focused psychotherapy, such as eye movement desensitization and reprocessing (EMDR), combined with a non-invasive brain stimulation technique, such as multifocal transcranial current stimulation (MtCS), could be an innovative adjunctive treatment option. This double-blind randomized controlled trial (RCT) analyzes if EMDR therapy is effective in the reduction of pain symptoms in FM patients and if its potential is boosted with the addition of MtCS.

**Methods:**

Forty-five patients with FM and a history of traumatic events will be randomly allocated to Waiting List, EMDR + active-MtCS, or EMDR + sham-MtCS. Therapists and patients will be kept blind to MtCS conditions, and raters will be kept blind to both EMDR and MtCS. All patients will be evaluated at baseline, post-treatment, and follow-up at 6 months after post-treatment. Evaluations will assess the following variables: sociodemographic data, pain, psychological trauma, sleep disturbance, anxiety and affective symptoms, and wellbeing.

**Discussion:**

This study will provide evidence of whether EMDR therapy is effective in reducing pain symptoms in FM patients, and whether the effect of EMDR can be enhanced by MtCS.

**Trial registration:**

ClinicalTrials.gov NCT04084795. Registered on 2 August 2019.

**Supplementary Information:**

The online version contains supplementary material available at 10.1186/s13063-021-05042-w.

## Background

Fibromyalgia (FM) is a chronic pain (CP) disorder characterized by generalized and widespread pain, sleep disturbance, fatigue, cognitive dysfunction, and affective symptoms. The average prevalence of FM worldwide is 2.7%, affecting mainly female patients [1]. Although the etiology of FM remains unknown, it is currently conceptualized as a disorder involving the sensitization of the central nervous system (CNS) and impairments in endogenous pain inhibitory mechanisms, with genetic, hormonal, and immunological factors playing a role [2]. Different risk factors associated with FM onset have been highlighted, including the presence of traumatic experiences such as sexual and physical abuse, chronic stress, and adverse lifetime events [3–5]. In fact, post-traumatic stress disorder (PTSD) is considered an important and frequently present comorbidity in patients suffering from FM [6, 7], although it is often not given priority as a treatment objective. While the mechanism which links adverse events to FM development is not fully understood, evidence shows that allodynia and hyperalgesia can be stress-induced [8], and psychological trauma is thought to lead to dysregulation of the hypothalamic-pituitary-adrenal (HPA) axis, linked to the aberrant cortisol secretion and epigenetic mechanisms [3, 4]. Therefore, psychological therapies focused on adaptive processing of traumatic experiences may improve both somatic and psychological symptoms and could be an innovative treatment option for FM.

Eye movement desensitization and reprocessing (EMDR) is a relatively new integrative psychotherapeutic approach developed by Francine Shapiro in the 1980s. It has been recognized by the World Health Organization as a first-line therapeutic tool for PTSD [9] due to its efficacy in reducing clinical symptoms, evidenced in several meta-analyses in both adults [10–14] and children and adolescents [15, 16]. Additionally, EMDR has shown its clinical applicability in the treatment of other pathologies, such as addictions [17], depression [18], anxiety [19], and CP [20]. In this last case, to date seven randomized controlled trials (RCT) have investigated the efficacy of EMDR in different conditions of CP: chronic pain [21, 22], migraine headaches [23], chronic back pain [24], pain due to rheumatoid arthritis [25], acute pain after abdominal surgery [26], and pain due to FM or diffuse chronic pain [27]. These studies showed EMDR significantly reduced pain levels in comparison with TAU [21, 24–26] and in comparison with other treatment options, for example, standard pharmacology [23], guided imagery [25], or eclectic therapy [27]. However, the studies on this topic are few and contain methodological biases, such as a lack of active control subjects, variability in PTSD and depression diagnoses, and the high associated comorbidity.

Given the complex etiology of FM, combining psychotherapy with other treatment options might increase the effectiveness of the treatment and maximize therapeutic success. Due to the key role that CNS sensitivity plays in FM, pioneering non-invasive brain stimulation (NIBS) techniques, such as transcranial direct current stimulation (tDCS), are increasingly being investigated, as these can modify neural activities related to pain. This neurophysiological technique represents a promising intervention option, given its capacity to modulate cerebral excitability in a safe, not invasive, and painless manner [28]. The principal mechanism of action of tDCS is a subthreshold modulation of neuronal membrane potentials, modifying cortical excitability and activity dependent on the current direction of flow through the target neurons [29]. Based on this, tDCS modulates spontaneous neuronal activity without generating activity changes in resting neuronal networks [30], its effects depending on the previous state of physiological basal activity of the region of interest [31].

There is increasing evidence which shows that, in addition to psychotherapeutic and pharmacologic interventions, tDCS is useful in treating FM and CP in terms of inducing pain relief and improving quality of life [32–46]. Interestingly, tDCS can also increase the therapeutic potential of other therapies when used in conjunction with them [47]. In this regard, a recent review has shown that the combination of tDCS with cognitive therapies, such as cognitive control therapy (CCT), increases its benefits when used for treating depressive disorders [47].

Taking into account the information presented above, we hypothesize that the combination of a trauma-focused therapy, such as EMDR, combined with tDCS targeted in the left dorsolateral prefrontal cortex (lDLPFC), could be a useful therapeutic option [48] for the treatment of patients with FM. The main reasons for selecting the lDLPFC are: (1) this region is critical in the regulation of the limbic system, implicated in emotion, which is hyperactivated in traumatized patients [49]; (2) it has been seen to be relatively hypoactivated in patients with depression [49–51], anxiety disorders [50, 51], and PTSD [52]; (3) there is a proved efficacy of lDLPFC tDCS in the treatment of resistant and non-resistant depression [47, 52], which suggests that FM patients will also benefit due to their comorbidities; (4) the lDLPFC also plays a central role in executive functions [53], and tDCS has the potential to improve cognitive functions associated with cortical plasticity, such as therapy-related learning processing [54].

Although in two studies it was found that the application of tDCS on the lDLPFC was generally less effective in reducing pain in FM patients compared to the application of stimulation on the primary motor cortex [35, 41], other studies were able to demonstrate a short-term efficacy in terms of pain and life quality [43], pain and fatigue [46], and pain and improvement in executive attention and orientation [42]. In all the studies mentioned, the stimulation applied over lDLPFC was anodal.

Regarding the mechanisms underlying the effects of DLPFC/tDCS, while no studies have specifically explored them, it is plausible that the cognitive and affective effects of DLPFC stimulation could be induced through the connections with the limbic system (fronto-limbic network) [55], whilst the effects on pain relief could be due to connectivity with the diffuse noxious inhibitory controls (DNIC) pathways that are involved in the inhibitory modulation of nociceptive input [56, 57].

Recently, multifocal transcranial current stimulation (MtCS) devices have been used to achieve more focal stimulation of specific cortical targets, because they use smaller electrodes compared to tDCS devices. Different methods have been designed to optimize the configuration of MtCS montage for stimulation of brain networks, represented by spatially extended cortical targets [58]. In this sense, MtCS could be more effective in modifying the functioning of the networks that are altered in patients with FM [55–57]. Previous studies with tDCS in FM have focused on the DLPFC; however, the effects of modulating the activity of this area with MtCS have not yet been explored.

## Methods/design

### Aims

The main objective of the study is to analyze whether EMDR therapy is effective in the reduction of pain symptoms in FM patients, and if its potential is boosted with the addition of MtCS. As secondary objectives, the study will analyze whether EMDR therapy is effective in reducing psychological trauma symptoms and comorbid symptoms of anxiety and depression, and in improving sleep quality and patient wellbeing, and if these effects are boosted by the addition of MtCS.

### Study design

Within a double-blind randomized controlled design, patients will be randomized to (1) Waitlist Condition, (2) EMDR + active-MtCS (20 sessions), or (3) EMDR + sham-MtCS (20 sessions). All subjects will continue to receive their treatment as usual (TAU), regardless of the group to which they have been assigned during the study. If a participant does not attend 3 sessions consecutively, she will be withdrawn from the study. Psychotherapists and patients will be kept blind for MtCS treatment conditions until the end of the trial, and raters will be kept blind to both EMDR and MtCS conditions. It is not possible for patients to be blind to the EMDR condition due to its use of bilateral stimulation. The experimental condition assigned to the participants will only be revealed if the patient abandons the study. Otherwise, the blind condition will be maintained until the end of the study. All patients will be clinically evaluated at baseline, at post-treatment, and at 6 months from post-treatment as follow-up.

**Fig. 1:**
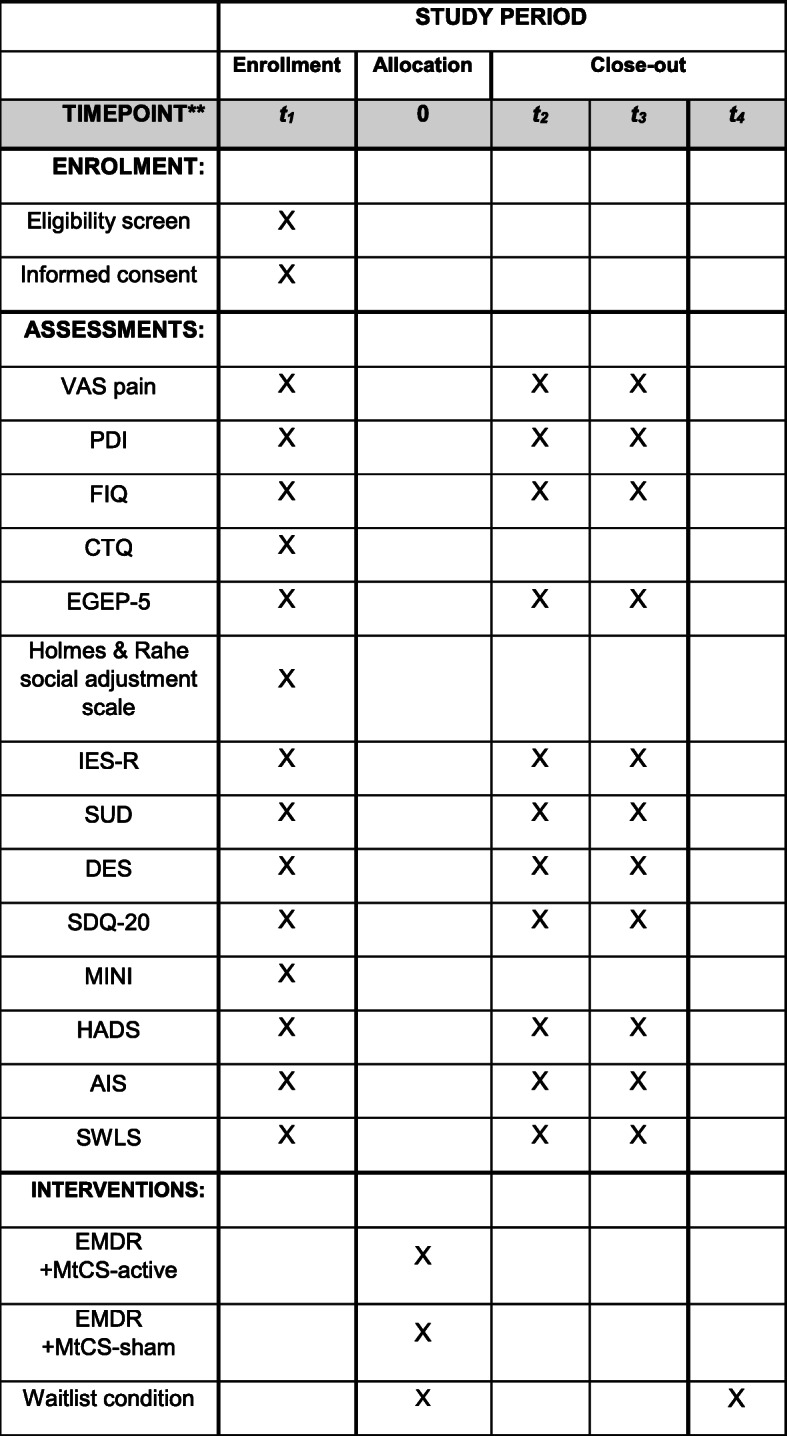
SPIRIT flow diagram: Schedule of enrollment, interventions and assessments. *t*_1_ = baseline evaluation; 0 = randomization process; *t*_2_ = post-treatment evaluation; *t*_3_ = follow-up evaluation; *t*_4_ = waitlist group participants receive treatment; VAS pain = visual analogue scale for pain; PDI = Pain Disability Index; FIQ = Fibromyalgia Impact Questionnaire; CTQ = Childhood Trauma Questionnaire; PTSD = Post-traumatic Stress Disorder; EGEP-5 = Evaluación General del Estrés Postraumático; IES-R = Impact Event Scale-Revised; SUD = Subjective Units of Distress; DES = Dissociative Experiences Scale; SDQ-20 = Somatoform Dissociation Scale; MINI = MINI International Neuropsychiatric Scale; HADS = Hospital Anxiety and Depression Scale; AIS = Athens Insomnia Scale; SWLS = Satisfaction With Life Scale

Figure 1 shows a flow chart of the progress of the study.

### Research setting

This multicenter collaborative project will involve the participation of the Centre Fòrum Research Unit of Parc de Salut Mar as the entity responsible for coordinating the study and carrying out the evaluations, the Rheumatology Department of Parc de Salut Mar for patient diagnosis and referral to the study, the Institut d’investigacions Biomèdiques August Pi i Sunyer (IDIBAPS) for randomization and data base management, and the Cognitive NeuroLab of Open University of Catalonia (UOC) for MtCS. External accredited EMDR psychotherapists, who have extensive experience and have received specific training for this study, will carry out the therapy, with supervision from the Centre Fòrum Research Unit (Barcelona, Spain). The study has been approved by the Ethics Committee of the IMIM, Parc de Salut Mar (2019/8772/I). All participants will sign the informed consent prior to enrollment in the baseline visit. Since this study involves a low-risk intervention, a Data Monitoring Committee will not be considered. Any deviation from the initial protocol will be communicated to the Ethics Committee through an official statement as well as to the Clinical Trials register.

### Participants

The patient sample will consist of 45 females who have been diagnosed by the Rheumatology Department of Parc de Salut Mar, Barcelona, Spain, through a clinical interview aligned with the 2016 American College of Rheumatology criteria for FM. Patients with this diagnosis will be referred from the Rheumatology Department, Anxiety Disorders Unit, Adult Mental Health Centers, and other departments of Parc de Salut Mar. When a participant meets the study criteria, she will be informed about the study and asked whether she would like to participate. Once she accepts, the raters will contact her to schedule the baseline visit and then she will be randomized to one of the groups. Inclusion criteria will be as follows: (1) aged between 18 and 70 years, (2) mean pain score of at least 4 on the visual analog scale for pain (VAS pain ≥ 4) in the 2 weeks preceding the clinical trial, (3) presence of one or more traumatic events causing current trauma-related symptoms (detection of at least one traumatic event using the EGEP-5 initial list of traumatic events, and the Impact of Events Scale-Revised ≥ 1 to assess the symptoms related to the traumatic event), (4) current clinical symptoms of depression and/or anxiety (Hospital Anxiety and Depression Scale ≥ 8), (5) stable medication regimen over the previous 2 weeks, and (6) met internationally established tDCS safety criteria [59]. Exclusion criteria will be as follows: (1) comorbid autoimmune or chronic inflammatory disease, (2) neurological or serious medical diseases, (3) bipolar disorder, schizoaffective disorder, or schizophrenia, (4) suicidal ideation, (5) previous EMDR therapy, (6) substance abuse/dependency within 1 month prior to participation (except for nicotine abuse/dependency), (7) pending FM-related litigation or disability, (8) metallic implants in the head, or (9) pregnancy.

### Randomization

The main analysis will be the comparison between patients assigned to EMDR vs those not assigned to EMDR. The secondary analysis, only amongst patients assigned to EMDR, will be the comparison between patients assigned to active-MtCS vs patients assigned to sham-MtCS. Therefore, the individuals will not randomly be assigned to one of the three arms. Instead, the patients meeting the inclusion criteria will be randomized twice: first to EMDR vs non-EMDR, and then those in the EMDR group to active-MtCS or sham-MtCS. For the sake of brevity, only the randomization to EMDR vs non-EMDR is described here, because the randomization to active-MtCS vs. sham-MtCS is identical. The variables used for the randomization will be age, educational level, and pain intensity score. The first two patients will be randomly allocated to EMDR with *p* = 2/3. For each subsequent patient, the following biased coin algorithm will be applied: if a group includes at least two more patients than it would have to have to maintain the ratio 2 EMDR / 1 control, the patient will be randomly assigned to the other group with *p* = 0.6. Otherwise, there will be a simulation of the new patient as it was allocated to EMDR and calculate the between-group standardized difference in pain intensity variable, then simulate that the new patient is allocated to non-EMDR and recalculate the difference, and finally randomly allocate the patient to the group associated to the smallest difference with *p* = 0.6. This strategy decreases prognostic imbalances between groups because it decreases differences in potential co-founders, yet still includes randomization.

Once the randomization group has been obtained, the principal investigator (PI) of the study will inform the participants accordingly. If the participants have been assigned to the EMDR group, the coordinator will also contact the therapist responsible of the treatment.

### Interventions

#### Eye movement desensitization and reprocessing (EMDR)

EMDR therapy will consist of a maximum of 20 individual 60-min sessions of psychotherapy, principally using the EMDR protocol for FM [60]. This protocol begins by gathering information about all aspects of the patient related to FM (phase 1) and helps create a hierarchy of the targets that are going to be processed during the sessions (phase 2). The following phases (3 to 8) follow the same steps as the EMDR Standard protocol [61]. The protocol is briefly described below:

1. Patient history: The therapist collects information about the patient’s biography in relation to the following aspects: history of FM, psychological trauma history, pain as a trauma, and pain triggers. These memories will be therapeutic targets in the following sessions. Treatment aims will be agreed between the patient and therapist.
2. Preparation: The patient will receive an explanation of the EMDR approach and how the therapy functions. The therapist will get to know the patient’s personal resources and check the patient’s preferences with regard to bilateral stimulation. If eye movements are not well tolerated, tapping or auditory tones will be used. Positive resources for emotional regulation and self-care will be installed.
3. Assessment: The therapist will help the patient focus on a traumatic event selected from the first phase. The patient will select the image that represents the most traumatic part of the event and the positive and negative cognitions associated with the memory, as well as the validity given to these cognitions using the Validity of the Cognition Scale (VoC; ranging from 1 signifying “completely false” to 7 signifying “completely true”). Emotions, sensations in the body, and the level of distress generated by the memory will also be registered by using the Subjective Units of Disturbance scale (SUD; ranging from 0 indicating “neutral or no distress” to 10 indicating “maximum distress”).
4. Memory desensitization: The patient will focus on the traumatic image and will associate it with the negative cognition, emotions, and bodily sensations reported in the previous phase. At the same time, the therapist will apply bilateral stimulation and the patient will observe any changes. After every set, the patient will inform the therapist about every change that has occurred. The role of the therapist will be to guide and accompany the patient during the processing until the SUD reaches 0.
5. Installing the positive cognition: The patient now focuses on the original memory and is asked to associate it with the positive cognition identified in the third phase. Bilateral stimulation will also be used to install the cognition.
6. Body scan: When the fifth phase is done, the patient will be asked to keep the memory and positive cognition in mind, and to scan their body for any sensations. If there is any negative sensation, bilateral stimulation will be applied until the sensation disappears. If there are only positive sensations, these will be installed through sets of bilateral stimulation.
7. Closure: When the session finishes, the therapist will explain that in the following days new material (such as new associations or memories) can arise, in which case the patient should register it for the following session.
8. Reevaluation: In the next session, the therapist will assess the state of the distress caused by the memory processed during the last session. If the memory has been correctly processed and no longer causes distress, the therapist will then proceed to treat other memories following the same protocol.

When a patient appears to suffer intense pain during the regular EMDR session, and the pain is threatening the patient’s processing, the CP protocol [62] will be used. Here, the target chosen is the current pain referred to by the patient. The main differences between the FM and the Pain protocols are the following: in phase 3, the patient must describe the pain felt and draw a representation of it, as well as assigning it personal characteristics; after phase 6, when improvements in levels of pain occur, the patient alongside the therapist will build a positive resource in order to reinforce the positive progress. It will then be followed by phases 7 and 8 of the standard protocol mentioned. Below is a brief description of the CP protocol:

1. Check that the patient’s pain is at a tolerable level by asking the patient to make a subjective assessment of their pain, evaluating their attitude to it, and ensuring that their pain is sufficiently controlled.
2. The medical diagnosis and the patient’s attitude toward it, including degree of acceptance, are reviewed.
3. The targets for EMDR reprocessing, and the treatment objectives (for example, pain relief or greater control over pain), are identified and put in order of priority and used to draw up a treatment plan. As pain is in many cases related, either directly or indirectly, to a traumatic or stressful event, these are treated first using the standard EMDR protocol explained above.
4. Next, each pain point is treated separately with the goal of helping the patient to relax and to notice changes in pain sensations. Bilateral stimulation is applied while the patient focuses on either current pain or a memory of pain. After each set, the patient explains their pain experience, and whether changes have occurred in the severity of the pain, its type, or where it is felt. The sets of bilateral stimulation are continued until the patient notices a positive change.
5. Finally, the patient is assisted in developing psychological pain management resources, achieved by the cognitive integration of the positive changes in pain sensations. First, the positive change is linked to an image, and this is reinforced through sets of bilateral stimulation. The patient then chooses a word to associate with the positive change, and this is reinforced through further sets of bilateral stimulation. The patient can then bring to mind the positive image and associated word and self-apply bilateral stimulation when they feel pain in the future, thus giving the patient pain management resources.

It is important to mention that this psychological intervention does not usually cause any risk to the health of the participants. However, due to remembering and reprocessing past traumatic experiences, it is possible that emotional discomfort will be felt during the evaluation and the therapeutic sessions. This discomfort usually disappears before the end of a session or, in exceptional cases, can also continue in the following day. If the discomfort continues, a new session with the patient should be immediately scheduled to assist her.

Should adverse effects other than those related to emotional discomfort from the therapeutic sessions occur, these shall be reported to the PI of the project. Additionally, if it is likely considered that EMDR was the cause, they will also be reported to the Ethics Committee department and relevant regulatory bodies, as required, indicating expectedness, seriousness, severity, and causality. However, as no problems that are detrimental to the participant are anticipated, no interim analyses or formal stopping rules have been planned.

Throughout the duration of the study, no participant may receive trauma-focused therapy sessions in parallel.

#### Multifocal transcranial Current Stimulation (MtCS)

**Fig. 2:**
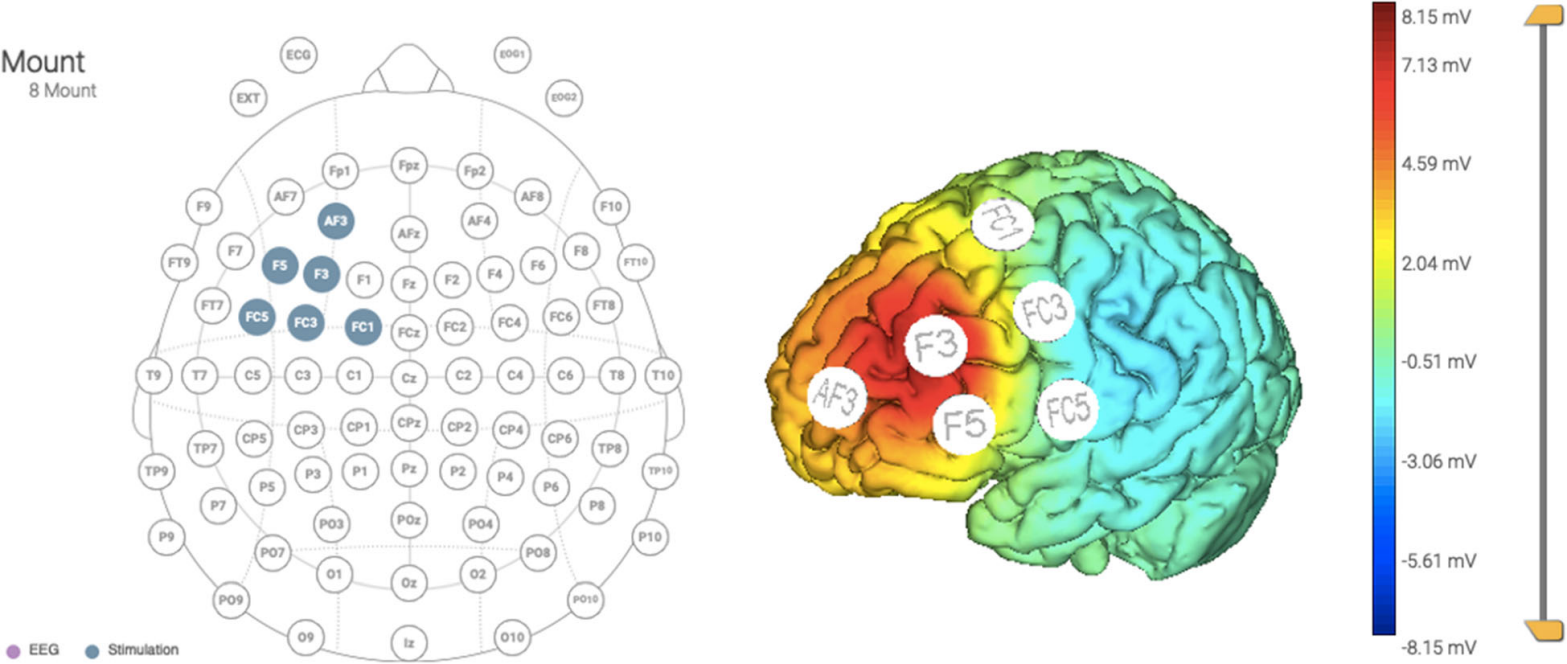
Multifocal transcranial Current Stimulation (MtCS) montage

Multifocal transcranial Current Stimulation (MtCS) montage (F3 anodal; AF3, FC1, FC3, FC5, F5, return) will be used with the anode over the lDLPFC. This montage, guided by StarStim® computational modeling data (see Fig. 2), was planned with the intention of enhancing the activity of the lDLFPC. Active stimulation will consist of 2 mA MtCS for 20 min applied immediately before EMDR sessions. The same protocol and montage will be used for sham stimulation, but the protocol will be implemented by ramping down (slowly) the current immediately after the ramp up period, and by ramping up (slowly) the current right before the final ramp down portion of the session. This way, the subject will feel the ramp up and ramp down events, but will not receive a significant dose of stimulation. Thus, the patient will believe she is being stimulated normally, but there should not be any real effects, in order to control for placebo effects of the MtCS treatment. The device used will be the StarStim®, which is a wireless hybrid EEG/tCS 8-channel neurostimulator system. StarStim® is currently classified as an investigational device under US federal law.

In the case of patients who have been prescribed medication with effects on the nervous system, such as antidepressants, anxiolytic drugs, anticonvulsants or atypical antipsychotics, an individualized follow-up of the clinical outcomes will be carried out to ensure that there is no interaction with the brain stimulation [28]. Although the application of MtCS is painless, in the event that a participant, due to her medical condition, feels marked pain or very significant discomfort due to the application of the stimulation and asks to stop the stimulation, this decision will be respected and the participant will receive EMDR treatment only. Again, if other different adverse effects than those mentioned above occur, they will be reported to the PI, and, in the event they are considered to be related to MtCS, also to the Ethics Committee department and relevant regulatory bodies as required, indicating the expectedness, seriousness, severity, and causality of the adverse effect. However, as has been mentioned previously, no problems that are detrimental to the participant are anticipated, meaning no interim analyses or formal stopping rules have been planned.

During the time of the study, no participant may receive MtCS sessions in parallel.

#### Waitlist condition

The patients allocated to this condition will follow their usual treatment without receiving any other additional therapy. Treatment as usual consists of regular visits with the rheumatologist, psychiatrist, and general practitioners, who are responsible for prescribing and monitoring the pharmacological treatment, principally in form of analgesics such as non-steroidal anti-inflammatory drugs, opioids, paracetamol, and/or gabapentin, but also antidepressant drugs and anxiolytics/hypnotics. Health psychoeducation by the nursing service and therapeutic physical exercise are also included in the waitlist condition. The patients from the waitlist condition group will be offered 10 sessions of EMDR therapy when their participation in the research project has finished. This has been decided for ethical reasons, but also with the aim that participants in the waitlist condition complete the study and thus the dropout rate in this arm can be reduced.

### Outcomes

Demographic and clinical variables will be collected through a clinical interview using the medical history of the patients and a specific Case Report Form (CRF) designed for the study which will include age, educational level, personal and family history, drug use, current pharmacological treatment and previous psychological treatment. We will also use the MINI International Neuropsychiatric Interview [63], Spanish validation [64] to explore the principal psychiatric disorders from Axis I of DSM-IV and CIE-10.

Pain intensity will be assessed using the following scales:

1. Visual Analog Scale for pain (VAS pain) [65]: The VAS Pain consists of a straight horizontal line, usually 10 cm long, anchored between 2 verbal descriptors: “No pain” on the left side and “Unbearable pain” on the right. Scores are interpreted as follows: no pain (0–2), mild pain (2–4), moderate pain (4–6), severe pain (6–8), and maximum pain (8–10). This measure assesses the intensity of the perceived pain over the last 2 weeks.
2. Pain Disability Index (PDI) [66], Spanish validation [67]. The PDI contains a list of 7 life categories that can be disrupted by chronic pain: family and home responsibilities, recreation, social activity, occupation, sexual behavior, self-care, and life-support activity. For each category there is a score from 0 to 10, higher scores mean greater disruption.
3. Fibromyalgia Impact Questionnaire (FIQ) [68], Spanish validation [69]. The FIQ is a 10-item self-administered scale for measuring physical impairment due to FM over the last week. Higher scores indicate greater impact in functioning.

Psychological trauma and trauma-related symptoms will be evaluated using the following scales:

1. Global Evaluation of Post-traumatic Stress (EGEP-5) [70]. The EGEP-5 is a 55-item clinician-applied scale to determine current PTSD diagnosis, based on DSM-V criteria. There are three different sections: presence of traumatic events, symptoms, and functioning. The scale can determine a diagnosis of PTSD, specifying the presence of dissociative symptoms (depersonalization and derealization) and delayed expression.
2. The Impact of Events Scale-Revised (IES-R) [71], Spanish validation [72]. The IES-R contains 22 items scored on a 5-point Likert scale, resulting in a score on 3 subscales (intrusion, avoidance and hyperarousal), with a total score ranging from 0 to 88. Higher scores represent greater distress. It measures the distress caused by a specific stressful life event over the previous 7 days.
3. The Holmes-Rahe Life Stress Inventory [73], Spanish validation [74]. This scale lists 43 possible stressful life events, each with a respective score. Global scores under 150 indicate low levels of stress, scores between 150 and 299 indicate a 50% risk of stress-related disorders and scores above 300 represent an 80% risk of suffering from stress [73].
4. The Childhood Trauma Questionnaire (CTQ) [75], Spanish validation [76]. The CTQ is a self-applied scale which includes a 28-item test that measure 5 types of childhood maltreatment: emotional, physical and sexual abuse, and emotional or physical neglect. A 5-point Likert scale (from 1 to 5) is used for the responses which range from “never true” to “very often true.” The final scores provide a severity score for each subscale from “none to minimal,” “low to moderate,” “moderate to severe,” and “severe to extreme.”
5. Dissociative Experiences Scales (DES) [77], Spanish validation [78]. The DES consists of 28 questions about different experiences related to dissociation, excluding when the subject has been intoxicated. This test is scored by summing the percentage score given in answer to each question (from 0 to 100) and then dividing by 28. A total score higher than or equal to 30 corresponds with high levels of dissociation.
6. Somatoform Dissociation Questionnaire 20 (SDQ-20) [79], Spanish validation [80]. The SDQ-20 is a 20-item self-report questionnaire measuring somatoform dissociation. Items refer to somatic symptoms and then ask if there is a known cause for them. The items are answered on a 5-point Likert scale and the symptoms with no known cause are summed to achieve the total score.

Anxiety and depression will be assessed using the following scale:

1. Hospital Anxiety and Depression Scale (HADS) [81], Spanish validation [82]. The HAD was created for detecting the presence of anxious and depressive disorders. It contains 14 items, 7 for each of the subscales (anxiety and depression), which can be rated from 0 to 3. A punctuation higher or equal to 11 indicates presence of affective disorder.

Quality of sleep will be assessed using the following scale:

1. Athens Insomnia Scale (AIS) [83], Spanish validation [84]. The AIS is a self-administered scale based on the ICD-10 criteria for insomnia. It measures sleep difficulties suffered over the previous three nights. It consists of 8 items evaluating sleep induction, awakenings during the night, final awakening, total sleep duration, sleep quality, wellbeing, functioning capacity, and sleepiness during the day. It is scored from 0 to 24 and higher scores mean greater difficulties.

Wellbeing will be assessed using the following scale:

1. Satisfaction With Life Scale (SWLS) [85], Spanish validation [86]. The SWLS is a 5-item self-administered scale measuring global cognitive judgment of satisfaction with one’s life. The items can be rated from 1 to 5, and lower scores indicate lower satisfaction.

All patients will be clinically evaluated at baseline/enrollment (*t*_1_), post-treatment (*t*_2_), and follow-up evaluation at 6 months from post-treatment (*t*_3_) (see Tables 1, 2, and 3). Personal participant data will be numerically coded and kept in a database in the Centre Forum Research Unit, who will be responsible for data maintenance and safety. Only the researchers involved in this trial will have access to this data. The database will contain the information of both the participants who finish the study as well as those who drop out, along with the corresponding causes for not completing the study. The Centre Forum Research Unit will be responsible for creating and maintaining the database.

**Table 1:** Measurements to evaluate pain and FM impact

Clinical variable	Measurement interview/self-report	***t***_**1**_ Baseline	***t***_**2**_ Post-treatment 6 months	***t***_**3**_ Follow-up 12 months
Pain intensity	VAS pain	x	x	x
Pain disability	PDI	x	x	x
FM impact	FIQ	x	x	x

*VAS pain* Visual Analog Scale for pain, *PDI* Pain Disability Index, *FIQ* Fibromyalgia Impact Questionnaire

**Table 2:** Measurements to evaluate psychological trauma symptoms

Clinical variable	Measurement interview/self-report	***t***_**1**_ Baseline	***t***_**2**_ Post-treatment 6 months	***t***_**3**_ Follow-up 12 months
Childhood trauma	CTQ	x		
PTSD	EGEP-5	x	x	x
Life events	The Holmes-Rahe Life Stress Inventory	x		
Trauma impact	IES-R	x	x	x
Distress associated to event	SUD	x	x	x
Dissociation	DES	x	x	x
Somatoform dissociation	SDQ-20	x	x	x

*CTQ* Childhood Trauma Questionnaire, *PTSD* Post-Traumatic Stress Disorder, *EGEP-5* Global Evaluation of Post-Traumatic Stress, *IES-R* Impact Event Scale-Revised, *SUD* Subjective Units of Distress, *DES* Dissociative Experiences Scale, *SDQ-20* Somatoform Dissociation Scale

**Table 3:** Measurements to evaluate clinical symptoms, insomnia, and quality of life

Clinical variable	Measurement interview/self-report	***t***_**1**_ Baseline	***t***_**2**_ Post-treatment 6 months	***t***_**3**_ Follow-up 12 months
Comorbidity	MINI	x		
Anxiety	HADS-A	x	x	x
Depression	HADS-D	x	x	x
Insomnia	AIS	x	x	x
Quality of life	SWLS	x	x	x

*MINI* MINI International Neuropsychiatric Scale, *HADS-A* Hospital Anxiety and Depression Scale-Anxiety, *HADS-D* Hospital Anxiety and Depression Scale-Depression, *AIS* Athens Insomnia Scale, *SWLS* Satisfaction With Life Scale

### Sample size calculation

The main tests of the study will consist of assessing whether patients assigned to EMDR show different levels in the pain intensity variable using a standard formula for two-tailed *t*-tests. The total sample size required to detect large to very large effect size differences (Cohen’s *d* ≥ 1) between two groups with a significance level of 0.05 and statistical power of 80% is 13 and 26. Assuming 15% dropouts, we will aim to randomize 45 patients, i.e., 15 + 30, so that after removing ~ 15% dropouts we would have approximately 13 + 26.

### Data analysis

The distribution of the sociodemographic and clinical variables between groups at baseline will be summarized by descriptive statistics. We will use *t*-tests to compare pain levels at post-treatment and follow-up between groups (Waitlist vs EMDR; active-MtCS vs sham-MtCS). In order to avoid regression toward the mean and confusion effects, baseline levels of pain will be added as covariates, as well as age, depression and anxiety severity and number of years of education. Due to the sample size, no further analysis is expected. The statistical software used for the analysis will be R. For the principal statistical analysis, an intention to treat (ITT) analysis will be used, and multiple imputation for losses at follow-up. The dataset analyses during the current study will be available from the corresponding author on reasonable request.

## Discussion

This scientific paper presents the first protocol of a double-blind RCT to investigate whether EMDR is effective in the treatment of FM and its comorbid symptoms, and if its potential is boosted with the addition of MtCS. Patients with FM can be a great clinical challenge for healthcare professionals, due to their high comorbidities and unexplained symptoms [87, 88]. The etiology of FM is poorly understood [3], consequently leading to an erroneous interpretation of the disorder and its manifestations, resulting in a pattern of excessive and non-beneficial therapy [89]. Patients usually feel frustrated about their treatment options [90] and dissatisfied with the support received: 38% of FM patients in Spain state that the public health system is the entity which gives them the least support, despite 72% of the FM patients only visiting doctors within this system. This lack of satisfaction with current diagnostic and treatment options could explain why 87% of FM patients prioritize scientific research as the means to a painless future [91]. It is the duty of researchers and clinicians to study precipitators and susceptible populations to build beneficial therapy protocols [4], and thus it is imperative to recognize the evidence showing the importance of the psychological trauma as a trigger in the development, maintenance, and chronification of FM, and provide suitable therapeutic options to address this.

This protocol is innovative in combining the EMDR approach for treating psychological trauma and reducing pain levels with MtCS. This approach should also help treat comorbidity, which, according to the results of recently published works, may also improve FM symptoms [89, 92–94]. The stress-related antecedents of FM include early-life traumas, PTSD, depression, anxiety, and major life stress [95]. Therefore, since traumatic events can predispose individuals to FM, mood, and anxiety disorders and influence mental and physical health [91, 94], the interventions appearing in this protocol seem beneficial.

Although there are several studies showing high comorbidity between FM and PTSD [4], very few studies have been carried out testing interventions targeting trauma-related comorbidity, and those which exist are of low quality [4], due to factors such as a lack of control subjects, variability in PTSD and depression diagnoses, and the high associated comorbidity [6]. Thus, this study will be a useful addition to the extant literature, and it will shed light on the possibility of developing a larger RCT using a new psychotherapeutic approach for the treatment of FM.

### Limitations

Some limitations of our trial have been taken into account. Firstly, the lack of control regarding drug treatment is a potential source of bias. To partly overcome this limitation, the current medication regimen should not be changed, as far as possible, once patients have been included in the study. Secondly, this study only includes females. Further studies would be needed to see if the findings of this study can be replicated in the much lower proportion of male FM patients. Thirdly, this is an exploratory and pragmatic trial with a relatively small size, meaning findings will later need to be replicated in a larger sample. Finally, another possible limitation could be the scalability of the MtCS treatment.

## Trial status

This is the first protocol version of the study. Recruitment began on 25 September 2019 and the plan was to finish this trial in December 2021. The COVID-19 pandemic, however, meant that the trial had to be suspended due to the impossibility of applying the intervention remotely. Once the current health situation permits it, our aim is to restart the trial from the beginning, to avoid bias in the results.

## Supplementary Information

**Additional file 1.** SPIRIT 2013 Checklist: Recommended items to address in a clinical trial protocol and related documents.

## Data Availability

Personal data of the participant will be numerically coded and kept in a database at the Centre Forum Research Unit. Only the researchers involved in the trial will have access to this data; however, the dataset analysis of the current study will be available from the corresponding author upon reasonable request. When the study finishes and results are obtained, we will send a copy of the article in Spanish to all, together with a plain language summary, as well as providing individual feedback about progress during the study if any participant requests it. The results of the study will also be published in peer review journals, and national and international conferences. The datasets analyzed during the current study will be available from the corresponding author on reasonable request.

## Abbreviations

FM: Fibromyalgia
EMDR: Eye movement desensitization and reprocessing
MtCS: Multifocal transcranial current stimulation
TAU: Therapy as usual
RCT: Randomized controlled trial
CP: Chronic pain
PTSD: Post-traumatic stress disorders
HPA: Hypothalamic-pituitary adrenal
NIBS: Non-invasive brain stimulation
tDCS: Transcranial direct current stimulation
CCT: Cognitive control therapy
lDPFC: Left dorsolateral prefrontal cortex
DNIC: Diffuse noxious inhibitory controls
CRF: Case Report Form
VAS: Visual analog scale
PDI: Pain Disability Index
FIQ: Fibromyalgia Impact Questionnaire
HADS: Hospital Anxiety and Depression Scale
AIS: Athens Insomnia Scale
EGEP-5: Evaluación Global de Estrés Postraumático-5
IES-R: Impact of Event Scale-Revised
CTQ: Childhood Trauma Questionnaire
DES: Dissociative Experiences Scale
SDQ: Somatoform Dissociation Scale-20
SWLS: Satisfaction With Life Scale
ITT: Intention to treat
LOCF: Last observation carried forward
